# 5-bp Classical Satellite DNA Loci from Chromosome-1 Instability in Cervical Neoplasia Detected by DNA Breakage Detection/Fluorescence *in Situ* Hybridization (DBD-FISH)

**DOI:** 10.3390/ijms14024135

**Published:** 2013-02-19

**Authors:** Elva I. Cortés-Gutiérrez, Brenda L. Ortíz-Hernández, Martha I. Dávila-Rodríguez, Ricardo M Cerda-Flores, José Luis Fernández, Carmen López-Fernández, Jaime Gosálvez

**Affiliations:** 1 Department of Genetics, Northeastern Biomedical Research Center, The Mexican Social Security Institute (IMSS), 64720 Monterrey, Mexico; E-Mails: brendal_o@hotmail.com (B.L.O.-H.); marthadavila@cibinmty.net (M.I.D.-R.); 2 Nursing Faculty, Autonomous University of Nuevo León, 64460 Monterrey, Mexico; E-Mail: ricardocerda_mx@yahoo.com.mx; 3 Section of Genetics and Research Unit, Hospital Teresa Herrera, Juan Canalejo University Hospital Complex, 15006 La Coruña, Spain; E-Mail: joseluis.fernandez@cog.es; 4 Unit of Genetics, Department of Biology, Autonomous University of Madrid, 20849 Madrid, Spain; E-Mails: mariadelcarmen.lopez@uam.es (C.L.-F.); jaime.gosalvez@uam.es (J.G.)

**Keywords:** cervical neoplasia, DBD-FISH, 5-bp classical satellite DNA

## Abstract

We aimed to evaluate the association between the progressive stages of cervical neoplasia and DNA damage in 5-bp classical satellite DNA sequences from chromosome-1 in cervical epithelium and in peripheral blood lymphocytes using DNA breakage detection/fluorescence *in situ* hybridization (DBD-FISH). A hospital-based unmatched case-control study was conducted in 2011 with a sample of 30 women grouped according to disease stage and selected according to histological diagnosis; 10 with low-grade squamous intraepithelial lesions (LG-SIL), 10 with high-grade SIL (HG-SIL), and 10 with no cervical lesions, from the Unidad Medica de Alta Especialidad of The Mexican Social Security Institute, IMSS, Mexico. Specific chromosome damage levels in 5-bp classical satellite DNA sequences from chromosome-1 were evaluated in cervical epithelium and peripheral blood lymphocytes using the DBD-FISH technique. Whole-genome DNA hybridization was used as a reference for the level of damage. Results of Kruskal-Wallis test showed a significant increase according to neoplastic development in both tissues. The instability of 5-bp classical satellite DNA sequences from chromosome-1 was evidenced using chromosome-orientation FISH. In conclusion, we suggest that the progression to malignant transformation involves an increase in the instability of 5-bp classical satellite DNA sequences from chromosome-1.

## 1. Introduction

Various risk factors have been correlated with the development of cervical intraepithelial neoplasia (CIN), suggesting that the human papillomavirus (HPV) type 16, 18, 31, or 33 (high risk) plays a pivotal role in this disease; however, additional chromosomal alterations seem to be necessary for the development and progression of CIN [1]. Increased genetic instability has also been considered a predisposing factor or primary event leading to neoplastic transformation.

Chromosomal anomalies in cervical tumors are either numerical, structural, or a mixture of both. Structural rearrangements of chromosome-1 (e.g., gains, deletions, translocations, and isochromosomes) have been described as the most frequent karyotypic changes in this type of tumor; for example, >95% of patients show rearrangements in this chromosome [2,3]. Aneusomy of chromosome-1 is strongly associated with infection high-risk HPV, and high-grade squamous intraepithelial lesions (HG-SIL) [4,5].

The DNA breakage detection/fluorescence *in situ* hybridization (DBD-FISH) technique is a new procedure that allows cell-by-cell detection and quantification of DNA breakage in the whole genome or within specific DNA sequences. Cells embedded in an inert agarose matrix on a slide are lysed to remove their membranes and proteins, and the remaining nucleotides are subjected to controlled denaturation with an alkali. The alkali transforms DNA breaks into restricted single-stranded DNA (ssDNA) motifs, which can be detected via hybridization with specific or whole-genome fluorescent DNA probes. As the number of DNA breaks increases in a target region, more ssDNA is produced and more probes hybridize, resulting in a more intense FISH signal, which can be quantified using image analysis systems [6–8]. Moreover, the alkaline treatment may break the sugar–phosphate backbone at abasic sites or at sites with deoxyribose damage, transforming these lesions into DNA breaks that are also converted into ssDNA. DNA damage levels may be a consequence of the torsional stress on DNA loops associated with tight chromatin packing, may vary between cell types in conventionally conformed genomes (e.g., sperm and lymphocytes) [9], and may change if the cell is under stress, such as in the presence of gamma irradiation [7].

Human satellite DNA includes a number of different tandemly repeated DNA sequence families that are organized in very large arrays. Alpha satellite DNA, which is located in the centromeric region of all chromosomes, is composed of monomers of 171 bp organized in higher-order repeated units. Their sequence divergence allows the isolation of specific subfamilies for each of the human chromosomes. Classical satellite 1, which is AT rich, is composed of two alternating units of 17 and 25 bp that are repeated tandemly. It appears to be located on pericentromeric regions of chromosomes 3 and 4 and on pericentromeric and distal short-arm regions of acrocentric chromosomes. The 5-bp classical satellites 2 and 3 contain sequence arrays based on tandem repetitions of variants of the “core” sequence 5′-TTCCA-3′ [10].

In human leukocytes, the DBD-FISH areas with a more intense background visualized using a whole-genome probe correspond to areas containing 5-bp satellite DNA sequences, with respect to the alpha satellite DNA region [11].

An increase in total DNA damage has been demonstrated in patients with cervical neoplasia using the micronuclei test [12], sister chromatid exchange [13], and the comet assay [14,15]. In a recent study, we used DBD-FISH to demonstrate a significant whole-genome increase in DNA damage according to cervical neoplastic development [16]. However, damage in specific DNA sequences in patients with CIN has not been studied.

The aim of this study was to evaluate the association between the progressive stages of cervical dysplasia and levels of specific DNA damage in 5-bp classical satellite DNA sequences from chromosome-1 in cervical epithelium and peripheral blood lymphocytes using DBD-FISH.

## 2. Results

Patients with HG-SIL exhibited more cells with numerical alterations (aneusomy and ploidy) compared with control individuals and patients with LG-SIL. The numerical alteration observed most frequently was tetraploidy (Table 1).

**Figure 1:**
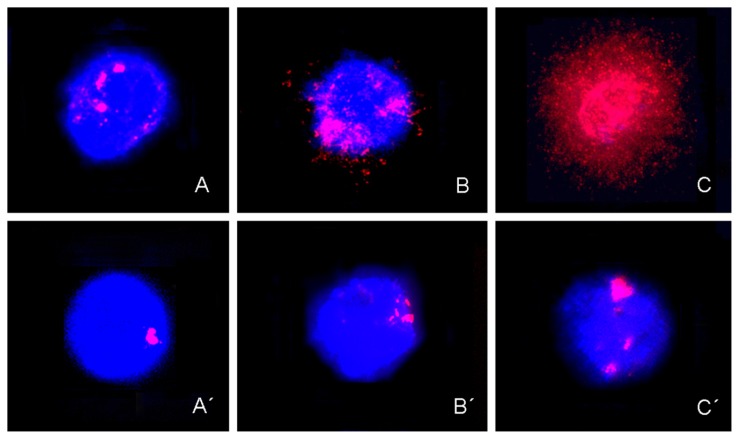
Representative set of cervical epithelium after DNA breakage detection/fluorescence *in situ* hybridization (DBD-FISH) in control women, with LG-SIL, and HG-SIL, using whole-genome probe (A to C respectively) and 5-bp classical satellite DNA sequences from chromosome-1 probes (A′ to C′ respectively) labeled with biotin (red) and counterstained with DAPI (blue).

**Figure 2:**
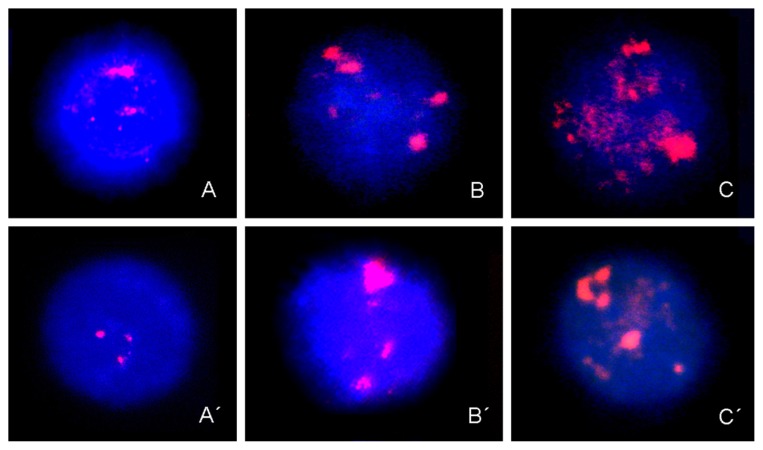
Representative set of peripheral blood lymphocytes after DBD-FISH in control women, with LG-SIL and HG-SIL, using whole-genome (A to C respectively), and 5-bp classical satellite DNA sequences from chromosome-1 probes (A′ to C′ respectively) labeled with biotin (red) and counterstained with DAPI (blue).

DBD-FISH performed under mild alkaline denaturing conditions led to the detection of DNA breakage in the whole genome and 5 bp classical satellite DNA sequences in chromosome-1. The labeling observed in control women represented the normal levels of DNA damage or constitutive DNA damage in cervical epithelia (Figure 1A,A′) and peripheral blood lymphocytes (Figure 2A,A′), respectively.

An increase in fluorescence signals in the whole genome or in 5 bp classical satellite DNA sequences in chromosome-1 was observed in cervical epithelia and peripheral blood lymphocytes from patients with HG-SIL (Figures 1C, 1C′, 2C, 2C′, respectively) *vs.* LG-SIL (Figures 1B, 1B′, 2B, 2B′, respectively).

As expected, the alpha satellite DNA region of chromosome-8 exhibited no significant differences according to diagnosis (data not shown).

The Kruskal–Wallis test revealed a significant increase in the hybridization signal of whole-genome DNA according to neoplastic development in cervical epithelia and peripheral blood lymphocytes (Table 2).

Regarding specific instability, a significant increase in the hybridization signal of 5 bp classical satellite DNA sequences from chromosome-1 according to neoplastic development was observed in both tissues (Table 2).

**Figure 3:**
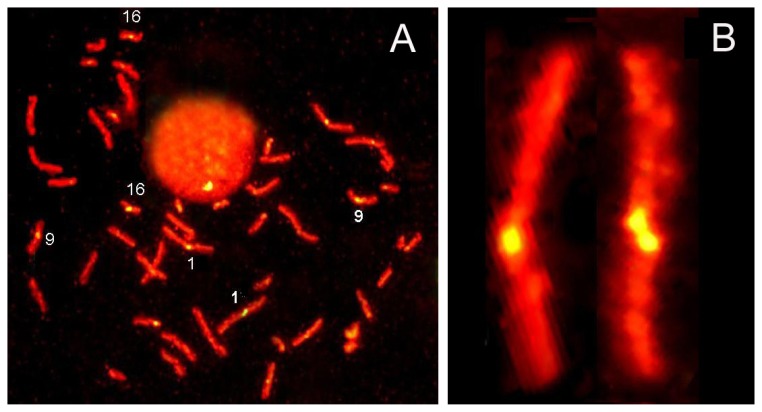
Chromosome-Orientation Fluorescence *in situ* Hybridization (CO-FISH) using Exo III DNA-selective DNA breakage in bromodeoxyuridine-treated cells and a human 5-bp repetitive DNA probe (chromosomes 1, 9 and 16) in patients with cervical neoplasia (**A**). Note the sister chromatid exchange affecting the specific DNA family in selected chromosome-1sequences (**B** right) respect to chromosome-1 without sister chromatid exchange in this region (**B** left).

The instability of 5 bp classical satellite DNA sequences from chromosome-1 in these patients was confirmed by chromosome-orientation fluorescence *in situ* hybridization (CO-FISH) (Figure 3).

All patients exhibited the presence of high-risk HPV; HPV-16 and HPV-52 were the most frequent viral types. Multiple infections were detected in 65% (13/20) of patients. All control women were negative for HPV (Table 3).

## 3. Experimental Section

### 3.1. Study Population

A sample of 30 women grouped according to disease stage and selected according to histological diagnosis [17] was included during 2011; 10 with low-grade squamous intraepithelial lesions (LG-SIL), 10 with high-grade SIL (HG-SIL), and 10 with no cervical lesions, from Unidad Médica de Alta Especialidad (UMAE) No. 23 of The Mexican Social Security Institute (IMSS) in Monterrey, Mexico. Written informed consent was obtained from all subjects and approval was given by the local Centro de Investigación Biomédica del Noreste (CIBIN), IMSS Ethical Committee.

The patients had received no previous chemotherapy or radiotherapy. The average age of patients and controls was 43.04 (range 31–55) and 45.51 (range 34–56) years, respectively.

### 3.2. Detection and Genotyping of HPV

HPV detection and genotyping were performed using an INNO-LiPA HPV kit (Innogenetics NV, Ghent, Belgium).

### 3.3. Cell Preparation

Cytological specimens were collected with a cytobrush from colposcopically abnormal areas (patients) and normal areas (controls).

The material was immersed in 5 mL of phosphate-buffered saline for transportation and was processed within an hour of sampling.

Peripheral blood samples from patients and controls were collected by venipuncture and transferred into EDTA-treated tubes. Peripheral blood leukocytes were isolated by centrifugation (35 min at 1300× *g*) in a Ficoll-Paque density gradient (Pharmacia LKB Biotechnology, Piscataway, NJ, USA). Leukocytes were suspended in 75 μL of low-melting-point agarose gel for embedding on slides.

Cell viability was measured using the trypan blue exclusion method. All samples had >85% viability.

### 3.4. Slide Preparation

Each slide was covered with 10 μL of 0.5% normal-melting-point agarose. These were immediately covered with 24 mm coverslips and maintained at room temperature for 5 min, to allow the agarose to solidify. This layer was used to promote the attachment of the second layer. About 10,000 cells from patients and controls were mixed with 76 μL of 0.5% low-melting-point agarose. After gentle removal of the coverslip, the cell suspension was pipetted rapidly onto the first agarose layer, spread using a coverslip, and maintained on an ice-cold flat tray for 5 min, to solidify. After the coverslip was removed, the third layer of 0.5% low-melting-point agarose (75 μL) at 37 °C was added, spread using a coverslip, and allowed to solidify on ice for 5 min.

### 3.5. DBD-FISH

DBD-FISH involves a protein-depletion procedure followed by treatment with an alkaline solution, to produce ssDNA. To deplete the proteins in cervical epithelium cells, and blood leukocytes, the slides were treated with a solution of 2 M NaCl, 0.05 M EDTA, 0.4 M Tris-base, and 1% SDS (pH 7) at 43 °C for 25 min. The slides were incubated horizontally, to avoid chromatin dispersion.

After the initial protein removal, the remaining nucleoids were washed in 0.9% NaCl for 10 min, to facilitate the final protein-removal step. To generate ssDNA, the protein-depleted slides were incubated in an alkaline unwinding solution containing 0.03 M NaOH and 1 M NaCl (pH 12.5) for 2.5 min at room temperature. After the sample was neutralized with 0.4 M Tris-HCl (pH 7.5) for 5 min, the nucleoids were washed in TBE buffer (89 mM Tris, 89 mM boric acid and 2.5 mM EDTA; pH 8.3) for 2 min. To stabilize the ssDNA, the slides were dehydrated in sequential 70%, 90% and 100% ethanol baths for 2 min each, and then air-dried. Biotin-labeled specific probes for the 5-bp classical satellite 2 of chromosome-1 (D1Z1) (Vysis Inc., Sunnyvale, CA, USA) were denatured and incubated overnight with the dried gels at room temperature. In other experiment, biotin-labeled specific probes for the alpha satellite DNA region of chromosome-8 (CEP-8, spectrum red) (Vysis Inc., Sunnyvale, CA, USA) was used as control.

To determine the total DNA damage (reference), a whole-genome DNA probe was produced from lymphocyte pellets using a DNA isolation kit for mammalian blood (Roche Diagnostics Corporation, Indianapolis, IN, USA). An aliquot (1 μg) of the DNA sample was labeled with biotin-14-2′-deoxyuridine 5′-triphosphate (dUTP) using a commercial nick-translation kit (Roche Diagnostics Corporation, Indianapolis, IN, USA). The slides were then washed twice at room temperature with 50% formamide and 2× SSC (pH 7) for 5 min, followed by 2× SSC (pH 7) for 3 min. The hybridized DNA probe was detected by incubation for 30 min with FITC-labeled avidin (1:400; Roche Diagnostics Corporation, Indianapolis, IN, USA). Finally, the slides were counterstained with 4′,6-diamidino-2-phenylindole (DAPI) (1 μg/mL) in Vectashield mounting medium (Vector Laboratories, Burlingame, CA, USA).

### 3.6. Image Analysis

All slides were analyzed on a digital image analysis platform based on *Zeiss Axiophot* (Carl Zeiss, Gottingen, Germany) fluorescence microscope equipped with three low-pass band filters to visualize green, red, and blue fluorescent emissions. The images were recorded using an Axiocam camera 16-bit black-and-white CCD camera in a 12-bit TIFF format. The integrated density (ID; segmented area of interest × gray-level values obtained after background subtraction) was calculated using the Image J 1.4.3.6.7 analysis software (National Institutes of Health, Bethesda, MD, USA, 2012). Fifty nuclei of cervical epithelium cells and fifty nuclei of peripheral blood leukocytes were examined for each individual. For determined ploidy and aneuploidy in smears cervical, and peripheral blood lymphocytes, FISH analyses using chromosome-1 (CEP-1, spectrum green), and chromosome-9 (CEP, spectrum orange) centromeric probes, were performed. One hundred cells/individual were analyzed. A cell was considered triploid or tetraploid if it was trisomic or tetrasomic for both chromosome-1 and chromosome-9. A cell was considered aneuploid if it had a chromosome complement that was greater than diploid but differed from a triploid or tetraploid cell (for example, three copies of chromosome-1 and four copies of chromosome-9). A cell was considered hypodiploid if any chromosome complement was less than diploid [18].

The sample size used for ANOVA comparison of means was estimated using the Minitab software (version 16; Minitab, Inc., State College, PA, USA, 2010) for DNA ssb damage (α = 0.05, 1 − β = 0.80, corrected sum of the squares of means = 371.85, and Sigma = 17.11) and DNA dsb damage (α = 0.05, 1 − β = 0.80, corrected sum of the squares of means = 171.17, and Sigma = 11.77). A value of *p* < 0.05 was considered significant for all tests.

### 3.7. Chromosome-Orientation Fluorescence *in Situ* Hybridization (CO-FISH)

CO-FISH was used to confirm the instability of 5-bp classical satellite DNA sequences from chromosome-1. Metaphase spreads were prepared from human lymphocyte cells, stained with Hoechst 33258 (Invitrogen), exposed to UV light (Stratalinker 1800 UV irradiator, La Jolla, CA, USA), and digested with exonuclease III (Promega M1811) to remove newly synthesized DNA strands. Hybridization and washing conditions were those described for FISH [19]. Chromosomes were hybridized with 5-bp classical satellite DNA loci probes at 37 °C overnight. Posthybridization washes were performed in 2× SSC at 42 °C. Finally, the slides were stained with FITC-labeled avidin (4 μg/mL, Fluorescein Avidin DCS; Vector Laboratories, Burlingame, CA, USA).

### 3.8. Statistical Analysis

Kruskal-Wallis test was used to investigate any differences in ID in the patients grouped according to disease stage. A value of *p* < 0.05 was considered significant. All analyses were performed using IBM SPSS for Windows 20.0 (IBM Corp., Armonk, NY, USA, 2011).

## 4. Discussion

Our results suggest that 5 bp classical satellite DNA sequences contain a large number of constitutive alkali-labile sites that are correlated with the grade of neoplastic progression. Although the causes of cervical carcinogenesis are not completely understood, the application of DBD-FISH using specific probes to detect DNA damage in genomic regions that are sensitive to destabilization may provide an essential tool for identifying cells that are at risk of progression.

Our findings showed the presence of a strong association between the increase in instability in 5 bp classical satellite DNA sequences from chromosome-1 and progressive stages in the development of cervical cancer. Given the high number of DNA breakages found in HG-SIL and the low number of DNA breakages detected in patients with LG-SIL, we suggest that the progression to malignant transformation involves an increase in the instability in chromosome-1. These results are in accordance with those of previous studies, which showed a significant positive correlation between numerical and structural aberrations in chromosome-1 and CIN lesions [5,20–23].

The presence of this instability in satellite DNA sequences in the genome can alter the organization of euchromatin and the nuclear environment. Methylation and histone modifications are often progressively compromised in cancer. Hypomethylation of satellite II involving the pericentromere of chromosome-1 has been reported in BRCA1, pancreatic cancer, and other epithelial cancers. Rearrangements in this region are the diagnostic tool for immunodeficiency centromeric region instability and facial anomalies.

Pericentromeric rearrangements and changes in nuclear positioning of chromosome-1 satellite II heterochromatin occur at an increased frequency compared with what is observed in other regions, suggesting the unique vulnerability of this block of satellite DNA. However, the genetic and epigenetic impacts of instability within these sequences are understudied, although they are a widespread phenomenon in cancer [24].

Conversely, it is possible that one or more tumor suppressor genes (*PAX7*, *FBG3*, *ARH1*, *NEK2*, *RGL* and *ARCH*) located on chromosome-1 are involved in the development or progression of cervical cancer [25].

The chromosomal instability observed in lymphocytes of women with cervical neoplasia is in agreement with the results of previous studies. Werkmeister *et al.* [26] reported that some genotoxic secretions are released by tumor tissues into the bloodstream, which might cause the significant increase in DNA damage [27], sister chromatid exchanges [13,28,29], micronuclei [12,30–32], and chromosome aberrations (chromatid-type, chromosome-type, and asymmetric aberrations) [32] observed in leukocytes.

The patterns identified in these cytogenetic studies indicate that chromosomal instability is a transient and chromosomally unstable intermediate in the development of cervical lesions. In this context, the mechanisms that may underlie the progressive increase in genetic instability in these patients seem to be related directly to HPV infection [33].

Previous studies have reported the presence of HPV-DNA in the serum and plasma of patients with cervical cancer, suggesting its potential use as a diagnostic marker [34]. Although the exact mechanism via which viral DNA is released into the bloodstream remains unclear, several pathways have been proposed, including necrosis and apoptosis [35].

We found that all patients with CIN presented with high-risk HPV infection. It may not have been possible to determine the association between HPV and DNA breaks in patients with neoplasia with or without HPV infection. However, in our previous study, we demonstrated this association via evaluation using the micronuclei test [36].

Women who develop cervical neoplasia may already be more prone to the development of DNA damage, which might explain why some women develop SIL after HPV infection and others do not.

Previous studies have shown an association between high-risk HPV and chromosomal instability [5,36]. In this context, the mechanisms underlying the increase in DNA damage in these patients might involve chromatin disorganization after HPV infection. Several studies have reported changes in chromatin organization during carcinogenesis and the subsequent association of distorted DNA-binding proteins with the nuclear matrix, which may have a functional role in chromatin organization and gene regulation [37].

Given our results and the multiple alterations in chromosome-1 reported previously in cervical neoplasia, further studies based on the combination of next-generation DNA sequencing, single-nucleotide polymorphism array analysis, and bioinformatics methods are needed to establish the threshold of 5 bp classical satellite DNA sequences in chromosome-1 that is associated with the chromothripsis phenomenon as a prevalent mechanism driving structural rearrangements in cervical cancer [38].

## 5. Conclusions

The instability of chromosome-1 defined as an increase in ID exhibited a stronger association with malignant transformation. However, further prospective studies are needed to determine the instability in 5-bp classical satellite DNA sequences in others chromosomes associated with cervical cancer (for example the chromosome-3), and to determine whether HR-HPV infection correlates with genomic instability, thereby establishing the usefulness of this test in the clinical management of women with cervical neoplasia.

## Figures and Tables

**Table 1:** Number of cells with numerical alteration (aneusomy and ploidy) observed in cervical epithelium and peripheral blood lymphocytes of control women, with low-grade squamous intraepithelial lesions (LG-SIL) and with high-grade SIL (HG-SIL).

Diagnosis	Aneusomy			Ploidy		
	
	Monosomy *	Trisomy **	Total	Triploidy	Tetraploidy	Total
Cervical Epithelium:						
Control	2	2	4	1	-	1
LG-SIL	3	4	7	1	1	2
HG-SIL	3	5	8	3	6	9

Lymphocytes:						

Control	-	-	-	-	-	-
LG-SIL	-	2	2	-	1	1
HG-SIL	3	2	5	-	2	2

* Monosomy of chromosome-1 or 9;

** Trisomy or tetrasomy of chromosome-1 or 9.

**Table 2:** Comparation of integrity density (ID) in whole genome and 5 bp classical satellite in chromosome-1, after fluorescence densitometry in cervical epithelium and peripheral blood lymphocytes of control women, with LG-SIL and with HG-SIL.

Tissue Type	Control (*n* = 10)		LG-SIL (*n* = 10)		HG-SIL (*n* = 10)		Kruskal Wallis	*p*
		
	Median	Mean Rank	Median	Mean Rank	Median	Mean Rank		
Cervical Epitelium:								

ID-WG	1.14 × 10^8^	5.80	6.12 × 10^9^	15.70	4.69 × 10^9^	25.00	23.79	0.000
Total NC	4600	13.25	4600	14.70	4600	18.55	3.525	0.172
ID Chromosome-1	1.65 × 10^4^	8.20	3.01 × 10^4^	15.50	8.68 × 10^4^	22.80	13.75	0.001
NC-1	200	14.50	200	14.70	200	17.30	1.04	0.594

Lymphocytes:								

ID-WG	3.58 × 10^4^	5.70	20.79 × 10^4^	15.90	165.99 × 10^4^	24.90	23.81	0.000
Total NC	4600	14.50	4600	15.95	4600	16.05	1.04	0.595
ID Chromosome-1	1.19 × 10^4^	5.50	9.89 × 10^4^	18.10	25.88 × 10^4^	22.90	20.84	0.000
NC-1	200	15.00	200	16.45	200	15.05	0.645	0.724

ID: Integrity Density; WG: Whole genome; NC: Number of Chromosomes; NC-1: Number of chromosomes-1; P: Probability.

**Table 3:** Human papillomavirus (HPV) Genotyping in cytologic specimens of women with LG-SIL and HG-SIL.

HPV GENOTYPING													
Diagnosis	16	18	31	33	39	44	51	52	54	58	68	70	82
LSIL	4	1	4	3	2	4	4	6	4	1	-	-	2
HSIL	6	2	2	-	4	2	4	3	3	1	1	1	2

TOTAL	10	3	6	3	6	6	8	9	7	2	1	1	4

HPV high risk: 16, 18, 31, 33, 39, 51, 52, 58, 82; HPV low risk: 44, 54, 68, 70.
